# The deleted in oral cancer (*DOC1* aka *CDK2AP1*) tumor suppressor gene is downregulated in oral squamous cell carcinoma by multiple microRNAs

**DOI:** 10.1038/s41419-023-05857-2

**Published:** 2023-05-22

**Authors:** Roberto Stabile, Mario Román Cabezas, Mathijs P. Verhagen, Francesco A. Tucci, Thierry P. P. van den Bosch, Maria J. De Herdt, Berdine van der Steen, Alex L. Nigg, Meng Chen, Cristina Ivan, Masayoshi Shimizu, Senada Koljenović, Jose A. Hardillo, C. Peter Verrijzer, Robert J. Baatenburg de Jong, George A. Calin, Riccardo Fodde

**Affiliations:** 1 Department of Pathology, Erasmus University Medical Center, Rotterdam, The Netherlands; 2 Department of Otorhinolaryngology and Head & Neck Surgery, Erasmus University Medical Center, Rotterdam, The Netherlands; 3 Department of Translational Molecular Pathology and Center of Department of Translational Molecular Pathology, and Center for RNA Interference and Non-Coding RNAs, The University of Texas MD Anderson Cancer Center, Houston, TX USA; 4 Department of Biochemistry, Erasmus University Medical Center, Rotterdam, The Netherlands; 5 Present Address: European Institute of Oncology IRCCS, Via Ripamonti 435, 20141 Milan, Italy; 6 Present Address: Caris Life Science, Irving, TX USA; 7 Present Address: Department of Pathology, Antwerp University Hospital, 2650 Edegem, Belgium

**Keywords:** Cancer genomics, Epigenetics

## Abstract

Cyclin-dependent kinase 2-associated protein 1 (*CDK2AP1*; also known as deleted in oral cancer or *DOC1*) is a tumor suppressor gene known to play functional roles in both cell cycle regulation and in the epigenetic control of embryonic stem cell differentiation, the latter as a core subunit of the nucleosome remodeling and histone deacetylation (NuRD) complex. In the vast majority of oral squamous cell carcinomas (OSCC), expression of the CDK2AP1 protein is reduced or lost. Notwithstanding the latter (and the *DOC1* acronym), mutations or deletions in its coding sequence are extremely rare. Accordingly, CDK2AP1 protein-deficient oral cancer cell lines express as much *CDK2AP1* mRNA as proficient cell lines. Here, by combining in silico and in vitro approaches, and by taking advantage of patient-derived data and tumor material in the analysis of loss of CDK2AP1 expression, we identified a set of microRNAs, namely miR-21-5p, miR-23b-3p, miR-26b-5p, miR-93-5p, and miR-155-5p, which inhibit its translation in both cell lines and patient-derived OSCCs. Of note, no synergistic effects were observed of the different miRs on the CDK2AP1–3-UTR common target. We also developed a novel approach to the combined ISH/IF tissue microarray analysis to study the expression patterns of miRs and their target genes in the context of tumor architecture. Last, we show that CDK2AP1 loss, as the result of miRNA expression, correlates with overall survival, thus highlighting the clinical relevance of these processes for carcinomas of the oral cavity.

## Introduction

Cyclin-dependent kinase 2-associated protein 1 (*CDK2AP1*) gene is a highly conserved tumor suppressor mapping to chromosome 12q24, originally identified and cloned from the Syrian hamster oral cancer model and named deleted in oral cancer-1 or *DOC1* [1]. The main tumor suppressive function originally attributed to *CDK2AP1*/*DOC1* lies in its role as a negative regulator of the cell cycle through the inhibition of cyclin-dependent kinase 2 (CDK2) and DNA polymerase alpha/primase during the S-phase [2]. However, more recent studies have revealed a novel role for CDK2AP1 as a core subunit of the Nucleosome Remodeling and Histone Deacetylation complex (NuRD), also likely to be relevant for its tumor-suppressing function [3]. NuRD recruitment drives extensive epigenetic reprogramming, including formation of inaccessible chromatin, H3K27 deacetylation, and recruitment of the Polycomb-repressive complex 2 (PRC2), followed by H3K27 methylation and H3K4 demethylation [3, 4]. Upon embryonic stem cell differentiation, activation of the mouse *Cdk2ap1/Doc1* gene mediates *Oct4* promoter methylation and downregulates its expression thus triggering differentiation. This silencing effect is dependent on the physical interaction between Cdk2ap1 and another core subunit of the NuRD complex, i.e., the methyl DNA- binding protein Mbd3 [5].

To date, which between the cell cycle- and epigenetic-related functions of *CDK2AP1* does represent its main tumor-suppressing activity, still remains elusive [6]. Recently, however, a study from our laboratory indicated that, in oral squamous cell carcinoma (OSCC), loss of DOC1 as an integral subunit of the NuRD chromatin remodeling complex, triggers the deregulation of a key functional hallmark associated to cancer progression and metastasis, namely epithelial-to-mesenchymal transition (EMT) and its reverse MET [7]. In the normal tongue epithelium, *CDK2AP1* expression follows an increasing gradient along the basal–squamous axis (i.e., it increases as cells start differentiating into squamous lineages), and its loss is associated with a substantial proportion of human oral squamous cell carcinomas (OSCCs) cases. CDK2AP1 was shown to direct the NuRD complex in the proximity of the promoters of important EMT transcription factors and master regulators such as *TWIST1*/*2*, *SLUG,* and *ZEB2*. As such, NuRD normally represses EMT in competition with the SWI/SNF complex that - on its turn - promotes EMT. These results suggest that SWI/SNF and NuRD function antagonistically to control the chromatin state and transcription of EMT-related genes [7].

Loss of expression of *CDK2AP1* has been observed in many different type of tumors, such as colon, gastric and esophageal cancers [8–10]. In all these studies, its repression correlates with adverse prognosis, tumor invasion, and metastasis. Although the majority of OSCC patients exhibits either complete loss or significant reduction of the protein encoded by *CDK2AP1* [7], the genetic mechanisms underlying its reduced or absent expression are still elusive as no point mutations nor deletions have been yet reported in its coding region [11, 12]. Two previous studies suggested that noncoding RNAs and in particular microRNAs (miR-21 and miR-205) could underlie the silencing of *CDK2AP1* expression in oral and laryngeal squamous cell carcinoma, respectively [13, 14]. MicroRNAs are short noncoding RNA transcripts capable of controlling gene expression by base pairing to specific sites at the 3’-UTR of target messenger RNAs, causing translational repression or degradation, depending on their binding affinity [15–17]. Here, by taking advantage of a combination of in vitro and in silico approaches, we have identified the main miRNAs responsible for *CDK2AP1* silencing in OSCC. Our results reveal that multiple miRNAs contribute to the loss of the tumor suppressor function of CDK2AP1 in OSCC patients in association with poor overall survival.

## Results

### Non-mutational mechanisms underlie the loss of CDK2AP1 expression in OSCC

**Fig. 1:**
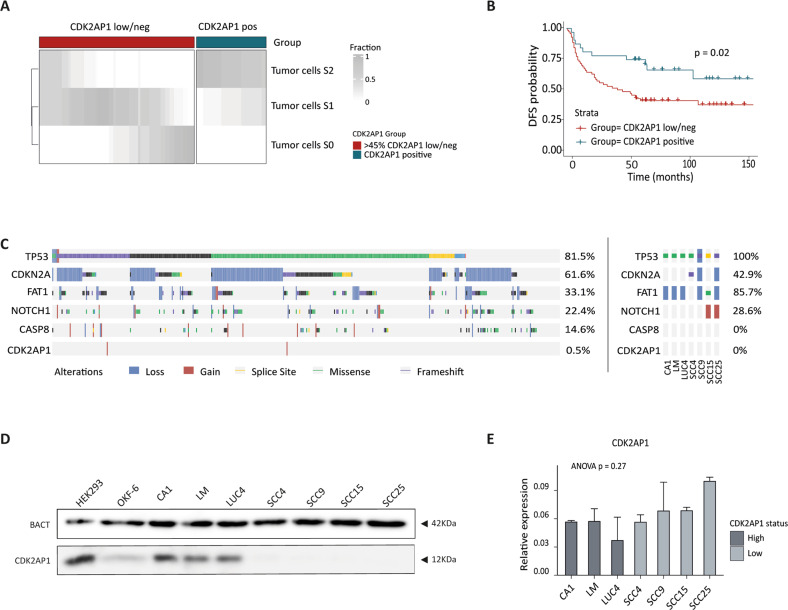
CDK2AP1 repression in OSCC is caused by non-mutational mechanisms and correlates with shorter disease-free survival. **A** Stratification of tongue carcinoma patients based on CDK2AP1 staining intensities. The 45% threshold of negative tumor cells was established based on the analysis shown in Supplementary Fig. 1. For each patient, the staining intensity fraction (S0, S1, and S2) is expressed according to the grayscale. Patients (*n* = 100) are from the RONCDOC cohort (see Table 1 and “Materials and Methods”). **B** Kaplan–Meier analysis of the disease-free survival (DFS) probability established on the RONCDOC cohort of oral squamous cell carcinoma of the tongue. The red line refers to patients with more than 45% of tumor cells negative for CDK2AP1 (low/neg), while the blue line indicates those with less of 45% of tumor cell negative for CDK2AP1 (positive). DSF probability for CDK2AP1-low/neg patients was significantly decreased when compared with CDK2AP1-positive tumors (Log-rank *P* = 0.02). **C** Graphic representation of the results of the somatic mutation analysis relative to 438 head and neck squamous cell carcinoma tumors (HPV infections excluded) from the TCGA dataset (left panel), and to the panel of OSCC cell lines analyzed by exome sequencing (right panel). **D** CDK2AP1 western blot analysis of the panel of OSCC cell lines. The 12 KDa CDK2AP1 protein is observed exclusively in the HEK293T, OKF-6 (here employed as positive controls), and in the CA1, LM, and LUC4 cell lines. The tongue OSCC cell lines (SCC4, SCC9, SCC15, and SCC25) do not express the mature CDK2AP1 protein. β-actin (BACT) was employed as a loading control. The blot shown here is a representative example of 3 independent experiments. **E** *CDK2AP1* RT-qPCR analysis of the protein-proficient (CA1, LM, and Luc4) and -deficient (SCC4, SCC9, SCC15, SCC25) oral cancer cell lines. The results are normalized based on GAPDH expression and are representative of three independent experiments.

While the loss of CDK2AP1 protein expression in oral squamous cell carcinoma has been previously reported [7, 18, 19], the underlying mechanisms are yet poorly understood. To further explore the relevance of CDK2AP1 loss in OSCC progression towards malignancy, we took advantage of a retrospective cohort of primary oral squamous cell carcinoma of the tongue (*n* = 100) collected between 2007 and 2013 at the Erasmus MC Cancer Institute and encompassing patients that received surgery as the primary form of treatment (Table 1). IHC analysis of CDK2AP1 expression in this cohort confirmed our previous report [7] in that a small percentage of the cases was either completely negative (∼10%) or positive (∼30%) for CDK2AP1 expression, with the vast majority of the tumors showing an admixture of negative and positive cells (Supplementary Fig. 1A–C). We then established two categories of CDK2AP1 immunoreactivity based on the staining intensity and an optimal threshold of 45% of cancer cells negative for CDK2AP1 (Supplementary Fig. 1D, E). Using this cut-off, disease-free survival negatively correlated with patients showing more than 45% of tumor cells negative for CDK2AP1 (Log rank *P* = 0.02; Fig. 1A, B).

**Table 1:** Retrospective cohort of primary oral squamous cell carcinoma of the tongue.

Clinicohistopathological characteristics	No. of patients		
		#	%
Sex			
	Male	59	59
	Female	41	41
Age at diagnosis (years)			
	Mean (range)	60.6 (21.0–90.0)	
Cancer stage (based on pTNM 8th edn. AJCC)			
	I	18	18
	II	30	30
	III	26	26
	IV	26	26
Treatment			
	Surgery	45	45
	Surgery and radiotherapy	55	55
CDK2AP1 status			
	<45% CDK2AP1 0 + 1	31	31
	≥45% CDK2AP1 0 + 1	69	69

Next, we interrogated the TGCA pan-cancer atlas dataset and in particular the mutation spectra relative to 438 patient-derived head and neck cancers not associated with HPV infection. As expected, the most commonly mutated genes were *TP53* (81.5% of the cases), followed by *CDKN2A* (61.6%), *FAT1*, and *NOTCH1* (33.1% and 22.4%, respectively). In contrast, the incidence of *CDK2AP1* genetic alterations was only 0.5%, mainly as the result of gene amplification events (Fig. 1C, left panel).

To further validate the low incidence of *CDK2AP1* mutations in oral squamous cell carcinoma, we established a panel of OSCC cell lines shown by western analysis to be either proficient or deficient for CDK2AP1 protein expression. As shown in Fig. 1D, the tongue squamous cell carcinoma cell lines SCC4, SCC9, SCC15, and SCC25 do not express the CDK2AP1 protein, whereas the OSCC cell lines CA1 (derived from floor of the mouth), LM (mandibular region of the mouth), LUC4 (floor of the mouth), and two non-cancerous immortalized control cell lines, namely OKF-6 (normal human oral keratinocyte) and HEK293T (human embryonic kidney), are positive for the 12 KDa protein (Fig. 1D). Direct mutation analysis of our panel of proficient and deficient cell lines by whole-exome sequencing (WES), and by interrogating the COSMIC database (https://cancer.sanger.ac.uk/cosmic) for SCC4, SCC9, SCC15, and SCC25, confirmed the absence of any *CDK2AP1* gene mutation in the cell line panel regardless of their positive/negative protein expression (Fig. 1C, right panel). Accordingly, RT-qPCR analysis of *CDK2AP1* transcriptional activity did not reveal any significant differences in expression values throughout the panel, irrespective of CDK2AP1 protein expression (ANOVA *P* = 0.27; Fig. 1E).

Altogether, these observations confirmed that, contrary to what its *DOC1* acronym would suggest, non-mutational and possibly post-transcriptional mechanisms underlie the silencing of CDK2AP1 protein expression in OSCC.

### Several miRNAs target the 3’ UTR of the CDK2AP1/DOC1 gene in OSCC

**Fig. 2:**
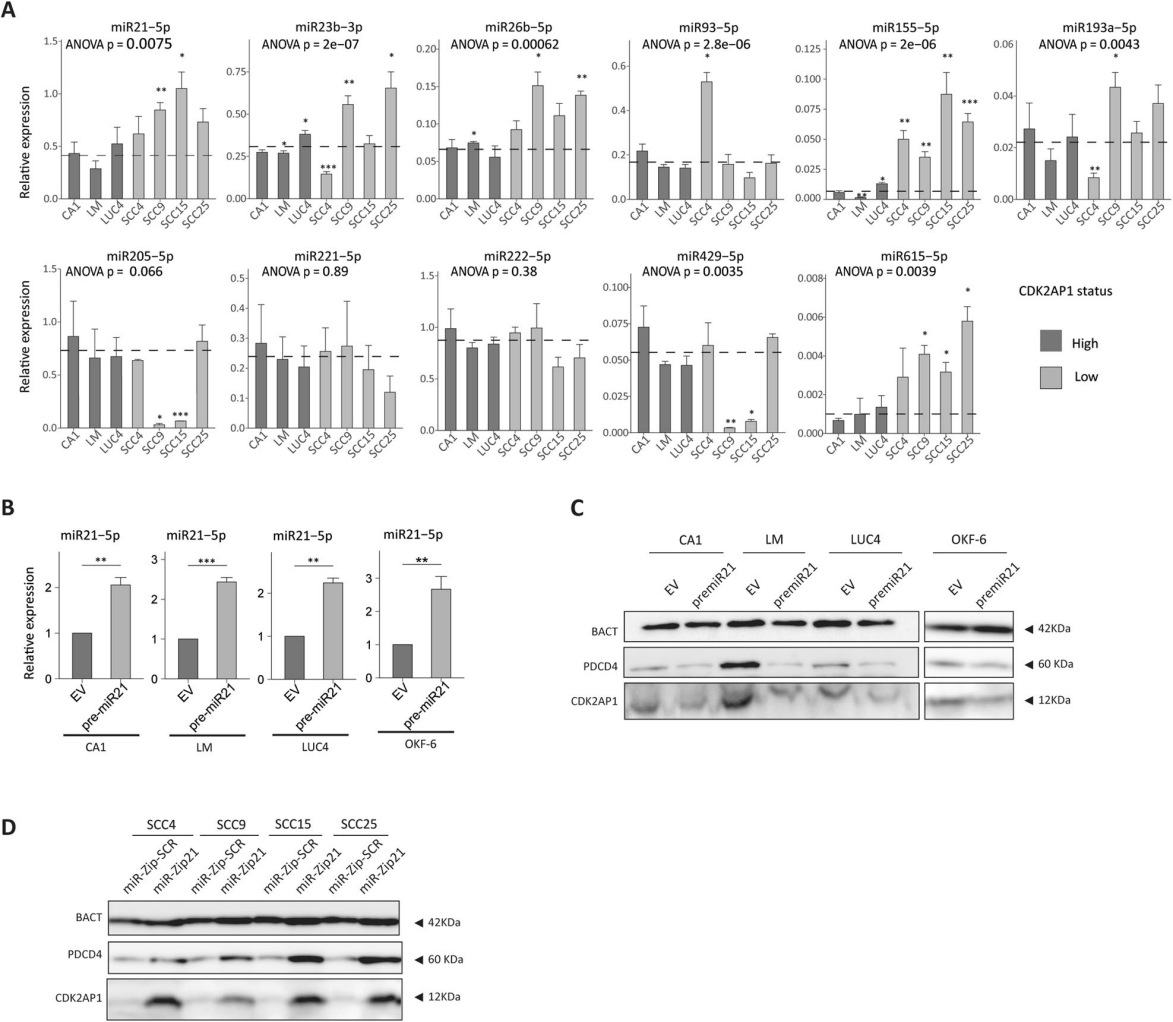
Identification of CDK2AP1-antagonist miRs in OSCC cell lines. **A** RT-qPCR expression analysis of the top 12 miR candidates arising from the microarray and in silico selection in CDK2AP1-deficient and -proficient cell lines. Of note, miR-592-5p expression levels are not shown due to undetectable expression. The dotted line indicates the average expression level of the analyzed miRNA in the proficient cell lines (CA1, LM, and LUC4). Experiments were performed in triplicate, and miRNA expression was normalized to that of snoRNA-U6. *P* values denote one-way ANOVA and one-sample *t* test against the mean of CDK2AP1-proficient cell lines (*P < 0.05; **P < 0.01; ***P < 0.001). **B** miR-21-5p RT-qPCR analysis of CDK2AP1-proficient cell lines upon transfection with either an empty (EV) or the pre-mir-21 PCDH-CMV lentiviral vector. Experiments were performed in triplicate and normalized to snoRNA-U6 expression. Fold change in miRNA expression was calculated relative to the EV-transfected cells for each experiment. One-sample *t* test *P* values: *P < 0.05; **P < 0.01; ***P < 0.001. **C** Western blot analysis of the consequences on CDK2AP1 and PDCD4 expression in the proficient cell lines transduced with an empty vector (EV) or with the pre-mir-21 PCDH-CMV lentiviral vector. Experiments were implemented in triplicate and β-actin (BACT) was employed as loading control. **D** Western blot analysis of the loss-of-function consequences of miR-21-5p inhibition on the CDK2AP1-deficient cell line panel. The SCCs cell lines were transfected with the MIRZIP lentiviral vectors targeting either miR-21-5p (mirZip-21) or a scrambled control sequence (miR-Zip-SCR). Experiments were performed in triplicate and β-actin (BACT) was employed as loading control.

As mentioned above, two studies have previously suggested the involvement of specific microRNAs, namely miR-21 and miR-205, in the silencing of CDK2AP1 expression in oral and laryngeal squamous cell carcinoma, respectively [13, 14]. In order to establish in a more comprehensive and systematic fashion the spectrum of miRs responsible for CDK2AP1 repression, a novel approach based on the integration of in silico analyses and in vitro assays was exploited (Supplementary Fig. 2). First, we performed a cell line-based miRNA microarray profiling of the CDK2AP1-deficient cell lines (SCC4, SCC9, SCC15, and SCC25). miRs whose expression levels were increased in the CDK2AP1-deficient lines were then further selected based on their affinity for the CDK2AP1–3’-UTR using the following databases: TarBAse v.8 [20], miRWalk2.0 [21], miRtarBase [22], and miRecords [23]. miRs with a final score of ≥7 (*n* = 12; see “Materials and methods”) were selected and validated by RT-qPCR in the CDK2AP1-deficient and -proficient OSCC cell lines (Fig. 2A). High degree of expression heterogeneity was observed even with miR-21, i.e., the most frequently overexpressed miR among human cancers, thus suggesting that the regulation of CDK2AP1 expression involves multiple noncoding RNAs. Based on these results, we selected five miRs, namely miR-21-5p, miR-26b-5p, miR-23b-3p, miR-93-5p, and miR-155-5p, whose expression was consistently and significantly increased in CDK2AP1-deficient cell lines, for subsequent functional validation. Of note, we did not include miR-193a-5p and miR-615-5p, whose expression was significantly increased in SCC9 (miR-193a-5p) and in SCC9, SCC15, and SCC25 (miR-615-5p) when compared with the CDK2AP1-proficient cell lines, due to the very-low-expression levels detected by RT-qPCR (Fig. 2A).

Next, we exploited the StarMIr tool [24] which allows the visualization of the interaction between each miR candidate and the *CDK2AP1–3’-UTR*. High-confidence structural predictions were obtained for all top 5 candidates, together with the identification of their seed sequences within the CDK2AP1–3’UTR sequence (Supplementary Fig. 3A, B). Of interest, two high-confidence predictions were made for the miR-155-5p interactor sites 1 (upstream) and 2 (downstream).

Altogether, these results indicate that multiple miRNAs contribute to suppress CDK2AP1 protein expression in our panel of OSCC cell lines and that 5 in particular appear to play a central role in this process.

### Functional validation of the newly identified CDK2AP1-antagonist miRs

We first analyzed the role of miR-21-5p, the top-ranked post-transcriptional regulator of CDK2AP1, by transfecting proficient cell lines with a pre-miR-21 expression vector (pCDH-CMV-MCS lentivector; System Bioscience). This approach ensures a more physiological maturation of the final miR [15] since overexpression of mature miRs can result in excessive levels (in the 100–1000-fold range) likely to cause off-target and artifactual effects [25]. The reverse approach was employed for the CDK2AP1-deficient cell lines, transduced with the miR-21 inhibitor (miR-Zip-21 lentivector; System bioscience).

Ectopic pre-mir-21 expression in the CDK2AP1-proficient cell lines (CA1, LM, and LUC4) as well as in the non-cancerous cell lines OKF-6, validated by RT-qPCR (Fig. 2B), results in a decrease of CDK2AP1 protein expression as shown by western blot analysis (Fig. 2C). Protein expression levels of PDCD4 (Programmed Cell Death 4), a well-established miR-21-5p target, were likewise decreased [26].

Vice versa, inhibition of miR-21-5p by miR-Zip-21 resulted in the rescue of CDK2AP1 and PDCD4 expression in the SCC4, SCC9, SCC15, and SCC25 cell lines (Fig. 2D). Notably, miR-21-5p inhibition in SCC9 resulted in consistent rescue of PDCD4, comparable with the other SCC lines, while CDK2AP1 re-expression was less prominent. The latter is likely to result from the action of miRs other than miR-21-5p, as also indicated by the significantly increased expression of the other candidates, i.e., miR-23b-3p, miR-26b-5p, and miR-193a-5p, in SCC9 (Fig. 2A).

**Fig. 3:**
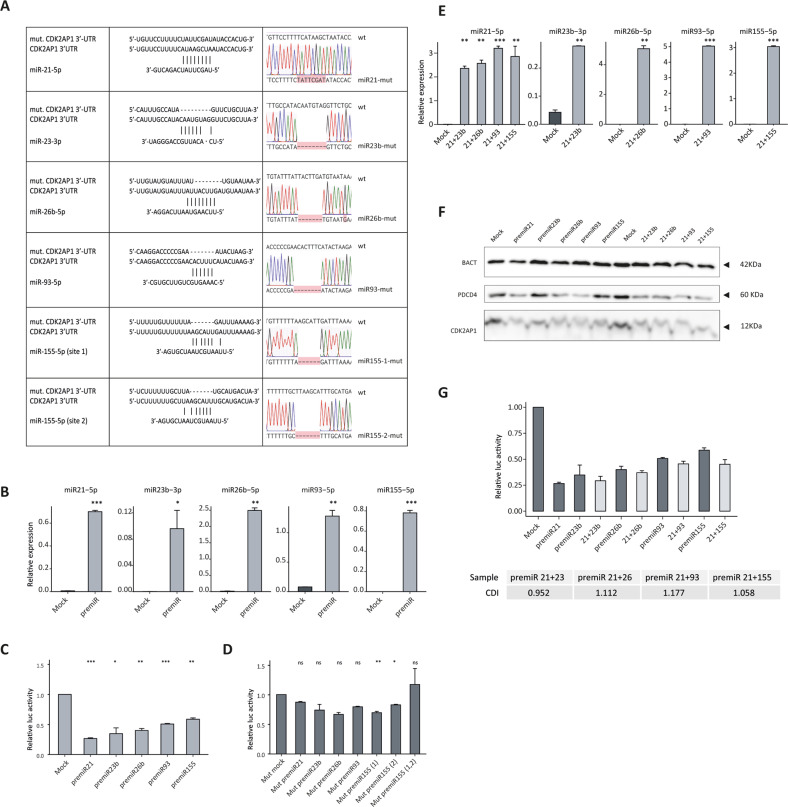
Luciferase reporter assay confirms miRs target specificity on the 3’-UTR of CDK2AP1. **A** Diagram illustrating the predicted interactions between the candidate microRNAs and the CDK2AP1–3’-UTR, as well as the mutations generated by site-directed mutagenesis (mut-CDK2AP1–3’-UTR) to abolish such interactions. **B** RT-qPCR analysis of mature microRNA expression in HEK293T cells transiently transfected with the pre-miR and with a mock empty vector as control. Experiments were performed in triplicate and normalized to snoRNA-U6 expression. Fold changes in miRNA expression were calculated relative to the EV-transfected cells for each individual experiment. One-sample *t* test *P* values: *P < 0.05; **P < 0.01; ***P < 0.001. **C** Relative luciferase activity measured upon ectopic expression of the miR-21, miR-23b, miR-26b, miR-93, and miR-155 pre-miRs vectors. Bars represent triplicate experiments normalized to mock-transfected cells. Mann–Whitney–Wilcoxon test *P* values: ^ns^*P* ≥ 0.05; *P < 0.05; **P < 0.01; ***P < 0.001. **D** Relative luciferase activity measured upon ectopic expression of the miR-21, miR-23b, miR-26b, miR-93, and miR-155 pre-miRs vectors encompassing the 3’-UTR mutated (mut) at the specific interactor sites. Experiments were performed in triplicate and normalized to mock-transfected cells. Student *t* test *P* values: ^ns^*P* ≥ 0.05; *P < 0.05; **P < 0.01; ***P < 0.001. **E** miR-RT-qPCR expression analysis of HEK293T transfected with an empty vector (mock) and with the pre-miR-21 expression vector in combination with the other candidate miRs (21 + 23b, 21 + 26b, 21 + 93, 21 + 155). Experiments were performed in triplicate and normalized to snoRNA-U6 expression (**P < 0.01; ***P < 0.001). **F** CDK2AP1 and PDCD4 western blot analysis in HEK293T cells transfected with an empty vector (mock) and with the pre-miR expression vectors relative to the individual candidates alone and in combination with miR-21 (21 + 23b, 21 + 26b, 21 + 93, 21 + 155). Experiments were performed in triplicate and β-actin (BACT) was employed as loading control. **G** Relative luciferase activity measured upon ectopic expression of miR-21, miR-23b, miR-26b, miR-93, and miR-155 alone and in combination with miR-21 (upper panel). Experiments were implemented in triplicate and normalized to mock-transfected cells. The Coefficient of Drug Interaction (CDI) values relative to each of the combination treatments are depicted in the bottom panel.

In order to ultimately validate the CDK2AP1-specificity of the selected miRs, the CDK2AP1–3’UTR full-length wild-type sequence (CDK2AP1–3’UTR, 754 bp; Supplementary Fig. 3B) was cloned in the pGL3-luciferase reporter vector (Promega). Mutant versions of the reporter vector (mut-CDK2AP1–3’UTR) were then generated by in vitro site-directed mutagenesis to carry specific alterations at the predicted miRNA-binding sites (Fig. 3A). The embryonic kidney cell line HEK293T was employed here due to its high transfection efficiency, to transiently express the newly generated pGL3-Luc vectors together with the corresponding miR expression vectors. Expression of the mature forms of each of the candidate miRs was confirmed by RT-qPCR (Fig. 3B) and luciferase activity was measured for each of the selected miRs. In the case of the wild-type pGL3-CDK2AP1–3’UTR vector, reduction of the relative luciferase activity compared to the mock control was detected for each of the 5 miRs ranging from 75% for miR-21-5p to 42% for miR-155-5p (Fig. 3C). The vectors encompassing targeted deletions of the specific seed sequences (pGL3-mut-CDK2AP1–3’UTR) abolished the miRs-driven reduction in reporter activity, thus confirming the 3’-UTR sequence specificity of their inhibitory effects (Fig. 3D). Notably, when multiple seed sequences for the same miRNA were identified in the 3’-UTR, as was the case of mir-155-5p, ablating only one of the two sites allowed only partial rescue of luciferase activity. Accordingly, when both predicted seed sequences were mutated, full rescue of luciferase expression was observed (Fig. 3D).

Altogether, these observations highlight the sequence specificity of the inhibitory effects exerted by the selected miRs on CDK2AP1 protein expression, and indicate that, notwithstanding the central role of miR-21-5p, multiple miRs target the CDK2AP1–3’-UTR and contribute to negatively regulate its translation. However, the co-expression of multiple miRs targeting the same 3’-UTR poses additional questions relative to their cumulative or synergistic effects on the inhibition of CDK2AP1 protein expression. To this end, we chose to transiently express the 5 miR candidates, individually and in different combinations, in the HEK293T cell line, and evaluate their effects on the inhibition of protein expressions by western blot as well as by luciferase reporter assay. As expected, transient expression of the five individual miRs led to reduction in CDK2AP1 and PCDC4 expression when compared to the mock control, with miR-21-5p showing the most pronounced effects, albeit not to a degree of full suppression (Fig. 3E, F). Instead, a more complete ablation of CDK2AP1 protein expression might be attained by the cooperation of multiple miRs. To test this hypothesis, we co-expressed miR-21-5p with each of the other miR candidates (Fig. 3E, F). As shown in Fig. 3G, we did not observe any significant effect or further reduction in relative luciferase levels upon co-expression of any of the four miRs with miR-21-5p. In order to quantitatively evaluate the degree of cooperativity among the different miRs, we calculated the Coefficient of Drug Interaction (CDI), defined as the quotient between the combination treatment (co-expression of miR-21-5p with each of the other candidates) and the average of the individual treatments. No evidence of significant synergism or even cumulative effects between any specific miR was found (Fig. 3G).

Overall, these results confirm the validity of our approach in that gain- and loss-of-function alterations in the newly identified miRs result in the down- and upregulation of CDK2AP1 expression. Moreover, the presence of seed sequences for the candidate miRs in the 3’UTR of the *CDK2AP1* gene which, when deleted, dramatically affect the capacity of the selected ncRNAs to modulate its expression, further validate the authenticity of our findings.

### Expression and relevance of the CDK2AP1-antagonist miRs in head and neck and oral squamous cell carcinomas

To assess the clinical relevance of the tumor-specific expression of the newly identified miRs, we analyzed miR-seq data obtained from patient-derived head and neck cancers, publicly available from the TCGA-HNSC database (https://portal.gdc.cancer.gov/projects/TCGA-HNSC), by integrating clinical follow-up information with miR expression profiles.

**Fig. 4:**
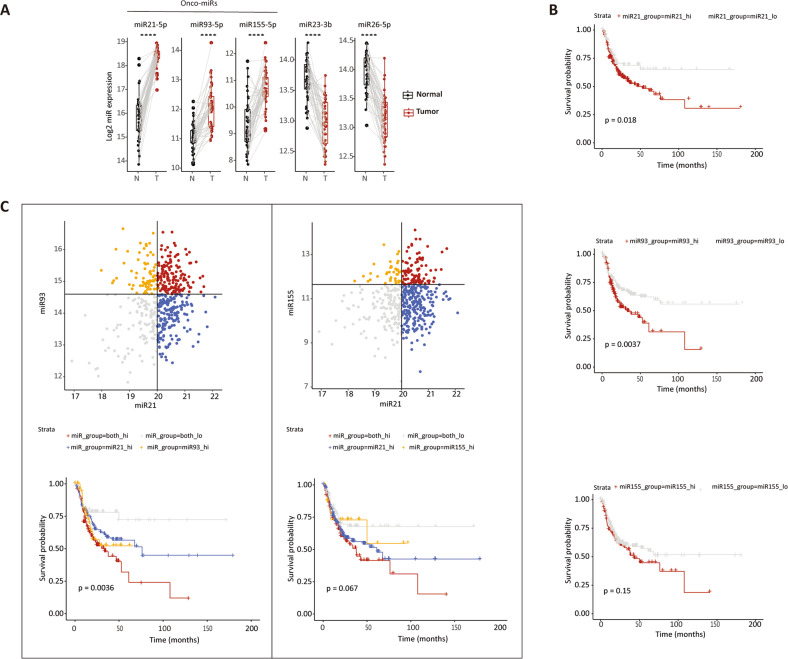
Identification and relevance of the CDK2AP1-antagonist miRs in primary tumors. **A** Comparative expression analysis of miR-21-5p, miR-23b-3p, miR-26b-5p, miR-93-5p, and miR-155-5p in patient-derived tumor and matched normal tissues (*n* = 45) from the TCGA-HNSC database (https://portal.gdc.cancer.gov/projects/TCGA-HNSC). Asterisks denote the significance of the *P* values by Wilcoxon-paired group comparison (****Padj <0.0001). **B** Kaplan–Meier analysis of the DFS probability relative to miR-21-5p (top), miR-93-5p (middle), and miR-155-5p (bottom) among patients from the above-mentioned TCGA-HNSC cohort. Significant associations are observed between miR-21-5p and miR-93-5p (*P* values obtained by Log-rank test). **C** Top panels: patients from the TCGA-HNSC database were subdivided into four groups based on the levels of miR-21-5p and miR-93-5p expression (left panel), and of miR-21-5p and miR-155-5p expression (right panel). Bottom panels: Kaplan–Meier analysis of DFS probability in the aforementioned groups. The combination of miR-21-5p with miR-93-5p improved on the separation of the two prognostic groups obtained with single miRs (*P* = 0.0036; Log-rank test). Combining miR-21-5p with miR-155-5p led to a slight improvement that, however, did not reach statistical significance (*P* = 0.067; Log-rank test).

We first stratified the miRs based on their tumor-specific expression levels compared with the matched normal tissues (Fig. 4A). Based on this analysis, we excluded mir-23b-3p and mir-26b-5p which showed significant downregulation in the tumor samples. Next, we stratified tumors with high- and low-miR expression. Kaplan–Meier analysis revealed that tumors with high expression of miR-21-5p and miR-93-5p share a worse overall DFS probability (*P* = 0.018 and *P* = 0.0037, respectively; Fig. 4B). In contrast, and in disagreement with previous reports [27–30], increased miR-155-5p expression does not affect survival probability (*P* = 0.15). The latter may be due to the heterogeneity of the head and neck cohort, encompassing tumors from different anatomical locations. Moreover, the miR-seq data were obtained from bulk preparations, inclusive of different cell types from the TME which may also express miR-155-5p and act as confounders [31, 32].

Next, we repeated the analysis by combining miR-21-5p expression with miR-93-5p and miR-155-5p. In the case of the miR-21/miR-93 combination, the difference in overall survival became significant when compared with cancers with increased expression of the single miRs (*P* = 0.0036; Fig. 4C). The same was not true for the miR-21/miR-155 combination (*P* = 0.067 vs. *P* = 0.018; Fig. 4C), although an improving trend, when compared with miR-21 alone, is observed. Therefore, the combination of miR-21-5p and miR-93-5p expression predicts at best overall survival in the analyzed cohort of head and neck cancers.

### IF/ISH analysis of tumor TMAs enables improved spatial characterization of the inverse correlation between CDK2AP1 and miR-21-5p expression

In order to better characterize the inverse relationship between CDK2AP1 and miR-21-5p expression as predicted by the results thus far, we performed a multiplex immunofluorescence (IF) and in situ hybridization (ISH) analysis of tumor tissue microarrays (TMAs) encompassing a retrospective cohort of primary oral squamous cell carcinoma of the tongue from patients that received surgery as the primary form of treatment (Table 1). In total, our TMAs included 432 tumor cores, derived from 144 OSCCs. From each tumor, three regions of interest were annotated by S.K. to be included in the TMA (see “Materials and methods”). In addition to CDK2AP1 and miR-21-5p, the tissue sections were stained with the 34BE12 antibody (Ventana) that recognizes several cytokeratins (i.e., CK-1, -5, -10, and -14) and distinguishes tumor cells from the surrounding stroma.

**Fig. 5:**
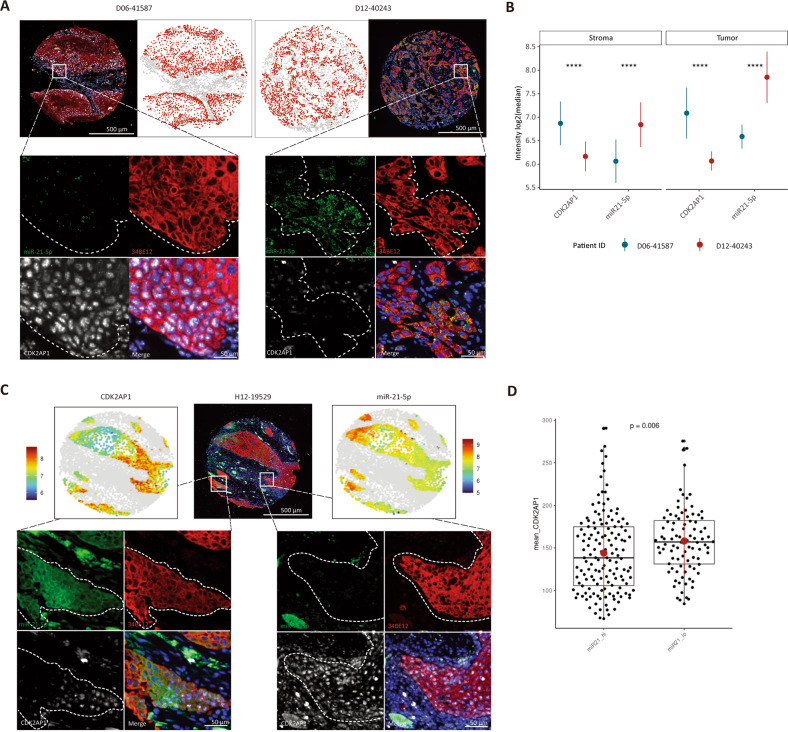
Multiplex IF/ISH analysis of TMAs enable improved characterization and spatial information relative to the miR-21-5p repression by CDK2AP1. **A** Representative images of CDK2AP1-positive (left) and -negative (right) TMA tumor cores featuring low and high expression of miR-21-5p, respectively. Next to the microscope images, digital reconstructions with tumor cells as red dots, and stromal cells as gray dots are depicted. In the lower panels, higher magnifications of the corresponding inlets are shown. **B** CDK2AP1 and miR-21-5p quantification of expression in the tumor and stroma components of the cores in (**A**). Asterisks denote the significance of the *P* values by one-sample *t* test (****P < 0.0001). **C**. Visualization of intra-tumor heterogeneity of CDK2AP1 and miR-21-5p expression levels in a TMA core and the digital reconstructions of the same microscope image. Opposite expression gradients of CDK2AP1 protein and miR-21-5p can be observed. In the lower panels, higher magnifications of the corresponding inlets are shown. **D** Box plot analysis of CDK2AP1 expression across miR-21-5p high and low groups (*P* = 0.006; paired *t* test).

Computational analysis of the digital TMA scanned images allowed us to derive signal intensities from every cell in each core, in the context of the spatial architecture of the tumors and the surrounding microenvironment. As depicted in Supplementary Fig. 4A, epithelial tumor cells (highlighted in red by 34BE12 expression and showed as red dots in the digital reconstruction) are clearly distinguished from the surrounding stromal cells (34BE12-negative and represented as gray dots. Representative images of CDK2AP1^pos^ and CDK2AP1^lo/neg^ tumors are depicted in Fig. 5A. The anti-correlation between CDK2AP1 expression in the nuclei and that of miR-21-5p in the cytoplasm is clearly visible in the higher magnification inlets of the cancer fields, and the digital representation of the expression densities (Supplementary Fig. 4B). The relative quantifications of the tumor and stromal compartments provide additional support for the antagonism between the tumor suppressor protein and the miR (Fig. 5B). Next, cores with less than 500 tumor cells (34BE12^pos^) were excluded and the relative abundance of miR-21-5p was established in the tumor and stromal compartments. As shown in Supplementary Fig. 4C, miR-21-5p expression appears increased throughout the TME. This approach highlights not only the inter-tumor heterogeneity but also the different CDK2AP1 and miR-21-5p expression patterns within each core when the microscope image is paired with the digital reconstruction across the TMA core (Fig. 5C).

Upon stratification of the TMA data based on mir-21-5p expression (mean_miR-21-5p > 150: high; mean_miR-21-5p < 150: low; Supplementary Fig. 4D), CDK2AP1 expression was found to significantly differ between the two groups (*P* = 0.006; Fig. 5D). However, it is noteworthy that considerable heterogeneity is observed again, suggesting that miR-21-5p is unlikely to be the only CDK2AP1 antagonist.

Taken together, these results confirm the anti-correlation between miR-21-5p and CDK2AP1 expression highlighting how the interaction between miRs and their gene targets can benefit from cell-type stratification and spatial information.

## Discussion

MicroRNAs are able to repress the expression of messenger RNAs bearing target sequences in their 3’-UTRs. Accordingly, the same mRNAs can interact with and be regulated by multiple miRs, orchestrating a complex regulatory network that controls gene expression.

After having confirmed the absence of somatic mutations affecting the *CDK2AP1* (*DOC1*) gene in OSCC, we identified multiple miRs that underlie the tumor-specific downregulation of its expression. Starting from an unsupervised genome-wide miRNA profiling of CDK2AP1-deficient OSCC cell lines, we shortlisted five potential *CDK2AP1*-regulating miR candidates, namely miR-21-5p, miR-23b-3p, miR-26b-5p, miR-93-5p, and miR-155-5p. Among these, miR-21-5p has previously been reported to target *CDK2AP1* in OSCC [13]. By means of in silico predictions, we identified their interaction sites in the 3’-UTR of *CDK2AP1* which were then validated by reporter assays. Gain- and loss-of-function analysis of each candidate miR further confirmed their role in downregulating CDK2AP1 protein expression. Notably, ectopic expression of each candidate miR proved insufficient to recapitulate the complete loss of CDK2AP1 expression observed in SCC lines, suggesting that multiple miRs may collectively contribute to its tumor-specific suppression. Nonetheless, we found no evidence of synergistic cooperation by combining miR-21-5p expression with the other selected miRs. Although miRNAs are believed to behave cooperatively in vivo, experimental demonstration of these alleged synergistic or cumulative effects has proven challenging [33, 34]. The implementation of single-cell miRNA sequencing techniques will address these issues in the near future [35, 36].

To analyze the clinical relevance of the selected CDK2AP1-antagonist miRs, we interrogated publicly available databases and validated the results by combining miR-FISH and CDK2AP1 IHC on tissue microarrays (TMAs) encompassing oral squamous carcinomas of the tongue. By taking advantage of this novel approach specifically designed to study miR/mRNA interactions, we provided additional and more specific evidence of the anti-correlation and spatial segregation between miR-21-5p and CDK2AP1 expression in patient-derived tumor sections. As also reported by Nouraee and colleagues in esophageal squamous carcinomas [37], tumor stroma contributes the most to miR-21-5p abundance in tongue SCC. This observation highlights how RNAseq data from bulk tumor resections may be misleading and of challenging interpretation when it comes to specific miRs and their role in regulating gene expression in different cell types. In view of the observation according to which miR/mRNA interaction does not always imply mRNA cleavage and its consequent degradation [38], our TMA-based ISH/IF multiplex approach allows to correlate expression levels of miRs and their target proteins in the context of tissue architecture. In the future, the integration of this approach with spatial transcriptomics will shed more light on the cellular and molecular mechanisms underlying the role played by miR-21-5p and other miRs in oral cancer progression, local dissemination and distant metastasis.

The oncogenic role of miR-21-5p is well-documented, since it is overexpressed in the majority of cancer types [39]. Significantly increased miR-21-5p expression levels have consistently been reported in tumors [29, 40] as well as in serum samples from OSCC patients [41] often in association with increased tumor keratinization and enhanced resistance to chemotherapy [42]. As such, miR-21-5p represents a predictor of local recurrence risk and overall survival in OSCC [43].

Similar to miR-21-5p, mir-93-5p is a well-known oncomiR found to be expressed in association with migration and invasion in a broad spectrum of cancer types, including stomach, pancreas, prostate, and lungs [44–47]. Likewise, miR-93-5p has also been reported to be co-expressed with EMT genes in head and neck SCCs, possibly promoting cancer progression and metastasis [40, 48]. Of note, increased levels of miR-21-5p and mir-93-5p have been detected in saliva from OSCC patients [49]. In view of the role of CDK2AP1 in the suppression of epithelial-to-mesenchymal transition in OSCC [7], it is plausible to think that the activation of the here described miRs counteract its TSG function at the invasive front. Our observation, according to which the combined expression of these miRs improves the separation of patients based on DFS, is of good auspices for future clinical value of multiple miRs as prognostic tool in oral cancer.

In parallel with mir-93-5p, miR-155-5p was also found to be significantly overexpressed in OSCC next to cancers of the lung, liver and breast [30, 50–52]. Notably, miR-155-5p was shown to be involved in chronic inflammatory processes [53], possibly of relevance in OSCC where chronic mucosal inflammation represents a key onset and progression risk factor [54]. However, although these studies have shown an association between miR-155-5p expression and adverse prognosis in OSCC and other malignancies [28, 30, 55], our in silico analysis suggests otherwise. This discrepancy could be explained by the different patient cohorts employed in the different studies. In an attempt to elucidate a common microRNA expression signature for a broad spectrum of human solid tumors (including lung, breast, stomach, prostate, colon, and pancreatic tumors), Volinia et al. confirmed that miR-155-5p, together with miR-21-5p and several other members of the miR-17-92 cluster form a robust oncogenic signature distinct from healthy tissue [56].

With regards to miR-23b-3p and miR-26b-5p, their expression is generally less abundant in tumors compared with normal tissues. This could imply that their detection in the panel of oral cancer cell lines may not be matched in vivo. Notwithstanding the latter, expression of these miRNAs have been reported in a broad spectrum of carcinoma types [57–59]. These observations highlight how the expression and therefore the effect of specific miRNAs can be tissue- and context-dependent. As such, we cannot exclude a role for miR-23b-3p and miR-26b-5p in OSCC until more in-depth and single-cell and/or spatial transcriptomic studies have been completed.

In conclusion, the results of the present study highlight the relevance of microRNAs in oral squamous cell carcinoma progression. “Non-mutational epigenetic reprogramming” has recently been established as a novel cancer-enabling characteristic among the hallmarks of cancer [60]. We have previously shown that CDK2AP1, as a member of the NuRD chromatin remodeling complex, plays a critical role in the “tug of war” between NuRD and SWI/SNF in the epigenetic regulation of the EMT master regulator genes *TWIST1/2*, *SLUG*, and *ZEB2* [7]. The present study adds another layer to these regulatory mechanisms where CDK2AP1 expression is controlled by multiple onco-miRs.

## Materials and methods

### Cell lines

The tongue-cancer-derived cell lines SCC4 (CVCL_1684), SCC9 (CVCL_1685), SCC15 (CVCL_1681) and SCC25 (CVCL_1682), obtained from ATCC, and the oral non-cancerous OKF-6 cell line, kindly donated by Dr. Conrado Aparicio (University of Minnesota), were cultured in DMEM F:12 with Glutamax™ (Gibco, 31331028) supplemented with 10% fetal calf serum (FBS; Thermo Fisher Scientific), 1% penicillin/streptomycin (Thermo Fisher Scientific, 15140122), and 400 ng/ml Hydrocortisone (Sigma, H0888). The CA1, LM and Luc4 cell lines, kindly provided by Dr. Adrian Biddle (Blizard Institute, Queen Mary University of London), were cultured as previously described [61, 62]. The HEK293T cell line, obtained from ATCC, was cultured in DMEM (Thermo Fisher Scientific, 11965092) supplemented with 10% FBS (Thermo Fisher Scientific), 1% penicillin/streptomycin (Thermo Fisher Scientific, 15140122), and 1% glutamine (Gibco, 25030024). All cells were maintained in a humidified atmosphere at 37 °C with 5% CO_2_. All cell lines have been authenticated using STR profiling and WES. Mycoplasma contamination testing was performed and the cells were proved to be mycoplasma free.

### Whole-exome sequencing of OSCC cell lines

DNA was extracted from the cell lines by the salting out procedure [63] and shipped to BGI genomics (www.bgi.com/global) for whole-exome sequencing, performed on the BGISEQ platform. The raw sequencing reads were processed using the nf-core sarek pipeline (version 3.0.2) [64] in tumor-only mode, using the GRCh38 as the reference genome. The pipeline included standard quality control, alignment, pre-processing, and variant calling steps based on GATK4 best practices [65]. The raw fastq reads have been deposited in the SRA database under accession code PRJNA962100.

Variants were called using Mutect2 and annotated using ANNOVAR [66] with the latest gene, region, and filter-based annotations available as of December 30, 2022. Only non-synonymous variants in exonic regions and variants affecting splice sites were considered. Variants occurring in 5 or more of the sequenced samples were excluded as likely arising from sequencing artifacts. Variants with a reported Minor Allele Frequency (MAF) ≥1% in either the 1000 Genomes [67], ESP6500 [68], or gnomeAD [69] databases were discarded as likely representing germline polymorphisms. Small insertions and deletions occurring in interspersed repeats and low-complexity DNA sequences were further excluded.

Copy number variation (CNV) inference was performed using CNVkit [70]. CNVs with a Log2 ratio >0.5 or <−0.5 and a *P* value < 0.05 were classified as gains and losses, respectively.

### miRNA array design and data analysis

The MDACCv5 miR arrays were generated as previously described [71] and analyzed according to the pipeline for Agilent miRNA arrays [72, 73]. Raw image data were processed into Matlab and not-annotated probes removed. The median foreground signal from each array was normalized using robust multichip averaging (RMA) [74]. Background correction was done with the Limma package in R. Duplicate probes were averaged and the data were standardized before multivariate statistical analysis. Hierarchical clustering, PCA, Partial Least Squares-Discriminant Analysis, and correlation computations were carried out in R (version 3.5.1) (http://www.r-project.org/). Experimentally validated miRNA-mRNA interactions were retrieved from several databases: miRWalk2.0 (http://mirwalk.umm.uni-heidelberg.de/), miRTarBase (https://miRTarBase.cuhk.edu.cn/), TarBase version 7 (http://diana.imis.athena-innovation.gr/DianaTools/index.php?r=tarbase/index) and miRecords (http://c1.accurascience.com/miRecords/). Target prediction was performed using miRWalk2.0 database, which hosts 12 existing programs to determine potential miRNA-binding sites of CDK2AP1. The Cancer Cell Line Encycolopedia (CCLE) database (https://depmap.org/portal/download/) was employed for miRNA expression in head and neck squamous cell carcinoma (HNSCC) cell lines.

We generated individual scores for each miRNA calculated by the sum of the following: (1) the expression based on the normalized array data and (2) the result from the miRNA interaction with mRNA analysis. Based on the resulting miRNA expression scores, the data were divided into four groups: 0 (if the mean value of the expression lies in the first quantile); 1 (if the mean value of expression lies between first and second quantile); 2 (if the mean value of expression is between second and third quantile); and the remaining values as 3.

The ranking criteria for the evaluation of miRNA-mRNA targets interaction were as follows: (1) the potential mRNA targets were not experimentally validated but predicted by less than 6 algorithms; (2) the mRNA targets were either experimentally validated (regardless of the target prediction results) or predicted by at least six distinct algorithms (out of 12 tested); (3) the mRNA targets were both experimentally validated and in silico predicted. The combinations of these two sets of scores gave score from 1 to 8.

### Structural prediction of putative microRNA-interactor sites

Structural predictions of the mature miR-seed sequence interaction were determined using STarMiR (Software for Statistical Folding of Nucleic Acids and Studies of Regulatory RNAs, https://sfold.wadsworth.org/cgi-bin/starmirtest2.pl) by including the microRNA IDs (hsa-miR-21-5p, hsa-miR-23b-3p, hsa-miR-26b-5p, hsa-miR-93-5p, hsa-miR-155-5p) and the NCBI reference ID for the target sequence for CDK2AP1–3’UTR (NM_004642).

### RTqPCRs

*CDK2AP1* gene expression analysis was performed by RT-qPCR using the Fast SYBR Green Master Mix (Thermo Fisher Scientific) on an Applied Biosystems StepOnePlus Real-Time Thermal Cycling system with three replicates per group. Relative gene expression was determined by normalizing the expression of each target gene to GAPDH (FW:5’-TCTAGACGGCAGGTCAGGTC-3’; REV: 5’-ACCCAGAAGACTGTGGATGG-3’). CDK2AP1 primers were: FW: 5’-GGCAACGTCTTCACAGTACC-3’; REV: 5’-CCAGTCCTCTAGCGTGAATG-3’.

For miRNA RT-qPCR, the TaqMan™ miRNA Assay kit (Applied Biosystems) and SsoAdvanced™ Universal Probes Supermix (Bio-Rad, cat number:1725285) were employed following the manufacturer’s instructions. Supplementary Table 1 summarizes the TaqMan™ probes employed for miRNA cDNA synthesis and RT-qPCR. Finally, RT-qPCR assays were carried out as duplicate reactions using the QuantStudio™ 12 K Real-Time system (Applied Biosystems). Relative miRNA expression was determined by normalizing the expression of each target miRNA to U6 snRNA. The results were analyzed using the 2-(ΔCt) method for group comparisons and the 2-(ΔΔCt) method [75] to evaluate the relative fold change to a reference sample.

### Western analysis

Cells were lysed in 10 mM Tris buffer pH7.5 containing 1% SDS, supplemented with complete™ Mini Protease Inhibitor Cocktail (Roche, 11836153001), and subjected to sodium dodecyl sulfate (SDS)- polyacrylamide gel electrophoresis (PAGE). Protein quantification was performed with the BCA Protein Assay Kit (Millipore); for each sample, 10 μg of protein was loaded. The membranes were incubated with primary antibodies against CDK2AP1 (1:500, Santa Cruz Biotechnology, sc-390283), PTEN (1:1000, Cell Signaling, 9559), PDCD4 (1:1000, Abcam, ab80590) and β-actin (1:2000, Cell Signaling, 4967), followed by polyclonal goat anti-mouse/rabbit immunoglobulins horseradish peroxidase (HRP)-conjugated secondary antibodies (Dako) diluted 1:1000. The signals were detected with Pierce ECT western blotting substrate (Thermo Fisher Scientific) using the Amersham 5 AI600 (GE Healthcare, USA).

### Luciferase reporters

Luciferase reporter vectors were constructed according to the New England Biolabs’ instructions (https://international.neb.com). The pGL3-Promoter vector (Promega), kindly donated by Dr. Laura Mezzanotte, was employed. The insert (3’UTR-CDK2AP1, NCBI accession number: NM_004642) was cloned downstream of the luciferase (*Luc2*) gene taking advantage of the XbaI and FseI restriction sites. The insert was PCR-amplified with primers designed to span the full-length 3’UTR (754 bp) and to introduce unique XbaI and FseI restriction cloning sites (Supplementary Table 2). Digestions were performed using 1 μg of DNA in a total reaction volume of 20 μl containing 1× CutSmart Buffer (New England Biolabs) and 1 U of each restriction enzyme (New England Biolabs) for 1 h at 37 °C. Digestions were quenched by heat inactivation at 65° for 20 min. Inserts were purified using the QIAquick PCR Purification Kit (QIAGEN, 28104). Ligation was performed using 500 ng of digested DNA and 0.5 U of T4 ligase in 1X T4 buffer (New England Biolabs) in a volume of 20 μl containing a mixture of vector and purified insert at 1:3 molar ratio for 2 h at room temperature. Chemically competent bacteria (Thermo Fisher, One Shot™ TOP10 Chemically Competent *E. coli*, C404010) were transformed according to the manufacturer’s protocol using 1 μl of ligation reaction. Plasmid DNA was extracted from positive colonies and sequenced to ensure the correct product was obtained using the primers listed in Supplementary Table 2.

### pre-miR expression vectors

pre-miR expression vectors were constructed according to the cloning workflow described by New England Biolabs (https://international.neb.com). The PCDH-CMV-MCS-miR-21 vector (SystemsBio), kindly donated by Dr. Joost Kluiver, University of Groningen, was employed to clone PCR-amplified inserts from genomic HEK293T DNA using the primers listed in Supplementary Table 2. Primers were designed to generate amplicons of 300–500 nt encompassing the pre-miR sequence of interest, and to introduce unique restriction cloning sites (BamHI and XbaI). All subsequent steps were performed according to the protocol described in the previous paragraph.

### Luciferase reporter gene assay

For this assay, 2 × 10^4^ HEK293T cells were seeded in each well of a 48-well plate. After 24 h, 500 ng of the reporter plasmid (pGL3-Promoter Vector, Promega) encompassing the CDK2AP1–3′-UTR sequence, together with 25 ng of Renilla plasmid (pRL-TK Vector, Promega), were co-transfected with 2000 ng of pre-miR expression vector (pCDH-CMV-MCS, Systembio). For pre-miR co-expression experiments, 1 μg of each expression vector was utilized. Plasmid co-transfections were performed in serum-free medium (Opti-MEM) in duplicate wells, using 2 μl of FuGENE® HD Transfection Reagent (Promega) for each μg of total plasmid DNA. Reporter assay was performed 48 h post-transfection using the Dual-Glo® Luciferase Assay System (Promega), according to the manufacturer’s protocol. Luminescence was detected by the GloMax® 96 Microplate Luminometer. Relative luciferase activity was determined by normalizing the Firefly reporter to Renilla luciferase activity.

### Site-directed mutagenesis of putative miRNA interacting sites

Primers were designed using the New England Biolabs Site-Directed Mutagenesis online design tool (https://nebasechanger.neb.com/; see Supplementary Table 2). The targeted sequence alterations designed to ablate the putative miR interactor sites were, for miR-21-5p, a 7-nt substitution with the complementary sequence, whereas for the remaining of miRs a 6–8 nt deletion spanning the interactor sites was introduced. Site-directed mutagenesis reactions were performed according to the PCR protocol (Q5 polymerase), using 20 ng of CDK2AP1–3’UTR luciferase reporter plasmid, supplemented with 5% DMSO in the case of the deletions. PCR products were digested with DpnI to eliminate the template DNA, and ligated in 1x KLD (Kinase-Ligase-DpnI) Reaction Buffer (Q5 Site-Directed Mutagenesis Kit, E0552S, New England Biolabs) for 30 min at room temperature. Chemically competent bacteria (Thermo Fisher, One Shot™ TOP10 Chemically Competent *E. coli*, C404010) were transformed according to the manufacturer’s protocol in 5 μl KLD reaction. Plasmid DNA was extracted from positive colonies and subsequently sequenced to ensure the correct product was obtained.

### Transient pre-miR and miR-Zip transfections

HEK293T cells were seeded in a 12-well plate at a density of 10^5^ cells per well. Cells in each well were transfected 24 h later with 2 μg of pre-miR expression vector (pCDH-CMV-MCS, Systembio) encompassing the pre-miR sequence of interest in serum-free medium (Opti-MEM) in duplicate wells, using 2 μl of FuGENE® HD Transfection Reagent (Promega) per μg of total plasmid DNA. Cells were harvested for RNA and protein 48 h post-transfection. From pre-miR combination experiments, 1 μg of each expression vector was utilized.

The OSCC cancer cell lines were infected with the lentiviral vectors described above in a 1:1 dilution with normal condition medium for 8 h. Cells were harvested for RNA and protein 48 h post-infection.

### Patient cohort, immunohistochemistry, and tumor tissue microarrays

Tissues from 100 primary oral squamous cell carcinoma of the tongue surgically removed between 2007 and 2013, were collected from the tissue bank of the Department of Pathology of the Erasmus Medical Center and recorded within the framework of the RONCDOC project, an initiative undertaken by the RWHHT (Rotterdamse Werkgroep Hoofd-Hals Tumoren). The cohort includes tongue squamous cell carcinoma (TSCC) removed by surgery as primary treatment at the Erasmus MC Cancer Institute. Cases with a previous history of head and neck cancer were excluded.

For all subjects included in this cohort, patient characteristics, comorbidity, TNM staging, treatment protocol, histopathological characteristics, recurrent disease and survival have been recorded. Tissue preparation and staining strategy were performed as described in Herdt et al. [76]. Briefly, using a microtome, consecutive 4-μm sections were cut from the formalin-fixed paraffin-embedded (FFPE) cancer tissues. Hematoxylin & eosin (H&E) as well as CDK2AP1 immunohistochemical staining (primary antibody 1:250, sc-390283, Santa Cruz) were evaluated and scored by a pathologist. The obtained data were then correlated with the clinical-pathological information included in the cohort.

The TMAs were obtained from the same FFPE blocks. Three regions of interest per block (one in the center of the tumor, two in the tumor border) were selected for sampling (1 mm diameter each) and included in the TMAs blocks.

Informed consent was obtained from all subjects.

### miRNA ISH by multiplex immunofluorescence

Four μm tissue sections on extra adhesive glass slides (Leica, Biosystems) were processed in the Discovery Ultra instrument (Ventana, Roche). The following automated Discovery Universal protocol was employed: tissues were preheated at 70 °C for 20 min, and de-paraffinized at 70 °C for 3 × 8 min. Pretreatment was performed with CC1 for 16 min (cat. no. 950–224, Ventana). One drop of DISC inhibitor (cat. no. 760–4840, Ventana) was applied and incubated for 12 min. The 3’ and 5’-DIG labeled miRCURY LNA miRNA Detection probes (hsa-miR-21, MiRCURY LNA miRNA YD00679870-BCG) was diluted in formamide-free MiRCURY LNA miRNA ISH Buffer (Qiagen cat. no. 339450) to a final 100 nM concentration, applied to the slides and incubated for 32 min. Denaturation was established at 90 °C for 8 min, followed by hybridization for 1 h. Slides were washed twice with SCC (DISCOVERY Ribowash 1× cat. no. 760–105, Ventana) and heated to 55 °C for 8 min. Slides were washed and heated again to 55 °C for 8 min. One drop of anti-DIG HRP enzyme conjugate (cat. no. 760–4822, Ventana) was applied and incubated for 16 min. Discovery amplification was performed by incubating one drop of DISC AMP TSA BF and one drop of DISC AMP H2O2 BF (cat. no. 760–226, Ventana) for 32 min. One drop of DISC anti-BF HRP (cat no. 760–4828, Ventana) was incubated for 16 min, followed by one drop of DCC (cat. no. 253-4971, Ventana). CC2 heat deactivation step was performed for 8 min at 100 °C followed by CC1 antigen retrieval for 24 min at 97 °C. CDK2AP1 at the dilution of 1:400 (Rabbit polyclonal antibody [7]) was incubated for 32 min at 37 °C followed by detection with omnimap Rb HRP (cat. no. 760-4311, Ventana) for 20 min and visualization with Cy5 for 8 min (cat. no. 253-4929, Ventana). CC2 heat de-inactivation step was performed for 8 min 100 °C followed by incubation of 34BE12 (1.4 μg/ml, cat no. 790-4373, Ventana) for 32 min at 37 °C followed by detection with omnimap Ms HRP (cat. no. 760-4310, Ventana) for 20 min and visualization with R6G (cat. no. 253-6003, Ventana) for 4 min. Slides were cleaned and coverslipped with DAP in Vectashield. Slides were scanned using the Zeiss Axioscanner 7.0 using ×20 magnification.

### TMAs bioinformatic data analysis

The multiplex TMAs were recorded with a Zeiss Axioscan 7 whole-slide scanner (Zeiss, Oberkochen, Germany) with filtercubes for DAPI, DCC, R6G, and Cy5 and a ×20 plan-apochromat NA 0.8 objective resulting in a pixel size of 0.345 μm. Whole-slide images were analyzed by VIS from Visiopharm (Visiopharm, Horsholm, Denmark, version 2023.11). First, the tumor regions were determined by an AI Deeplab algorithm based on the R6G signal. In tumor and non-tumor regions nuclei and cytoplasm were detected by a second AI U-net algorithm based on the DAPI signal. For every cell, the *x*- and *y*-coordinates were determined, and intensities (mean, median, SD) of the miRNA- and CDK2AP1-signal (DCC and Cy5) in the nucleus and cytoplasm were measured.

Next, matrices containing features from the different fluorescent channels were imported in R for downstream analysis. Matrices from all cores (N = 432) were merged, and outliers were filtered from the data by excluding cells with *z*-scores >3 from the median intensity channels (2.9% of cells). Inter-core comparative analysis was performed by averaging the median cellular intensities (median cytoplasmic miR-21, median nuclear CDK2AP1) within the tumor compartment for cores with at least 500 tumor cells. Cores were ranked based on their inverse Pearson correlation of miR-21 and CDK2AP1. For visualization of TMA cores, median intensities were log2 normalized and cells were plotted using their respective x and y-coordinates based on the center of their cytoplasm.

### TCGA repository data analysis

Publicly available RNA and miRNA sequencing data of head and neck squamous cell carcinoma patients from the cancer genome atlas (TCGA-HNSC cohort, *N* = 547 sample) [77] was retrieved from the NCI Genomic Data Commons (GDC) and LinkedOmics repositories [78]. Processed copy number variation (CNV) and exome sequencing data were obtained from cBioPortal [79]. Tumors associated with HPV infections (*n* = 73) [80] were excluded from the analysis. The miRNA sequencing counts were normalized with variance stabilizing transformation (VST) from the DESeq2 package. Survival analysis was performed with the survival and survminer packages using the LogRank test on the disease-free survival (DFS).

### Statistical analysis

Results are shown as means of three biological replicates, with error bars representing the standard error of the mean (SEM). Details of each statistical test are indicated in the figure legends. All statistical tests and graphs were executed using the R software package.

## Supplementary information

Supplementary Material

Supplementary Table 3

Original Data File

Reproducibility Checklist

## Data Availability

All data used in this study are included in this published article (and its Supplementary information files).
