# Supplementary material for: The deleted in oral cancer (DOC1 aka CDK2AP1) tumor suppressor gene is downregulated in oral squamous cell carcinoma by multiple microRNAs
Source: Cell Death Dis. 2023 May 22;14(5):337. doi: 10.1038/s41419-023-05857-2 (PMC10202934; doi:10.1038/s41419-023-05857-2)
Supplement: Supplementary file 1 — Supplementary Material [file 41419_2023_5857_MOESM1_ESM.docx]

**Supplementary file for**

**The deleted in oral cancer (*DOC1* aka *CDK2AP1*) tumor suppressor gene is downregulated in oral squamous cell carcinoma by multiple microRNAs.**

Roberto Stabile^1^, Mario Román Cabezas^1^, Mathijs P. Verhagen^1^, Francesco A. Tucci^1#^, Thierry P.P. van den Bosch^1^, Maria J. De Herdt^2^, Berdine van der Steen^2^, Alex L. Nigg^1^, Meng Chen^3^, Cristina Ivan^3+^, Masayoshi Shimizu^3^, Senada Koljenović^1$^, Jose A. Hardillo^2^, C. Peter Verrijzer^4^, Robert J. Baatenburg de Jong^2^, George A. Calin^3^, and Riccardo Fodde^1*^.

*to whom correspondence should be addressed at [r.fodde@erasmusmc.nl](mailto:r.fodde@erasmusmc.nl)

This files includes:

Supplementary Fig, 1-4

Supplementary Tables 1 and 2

**Supplementary Figures**


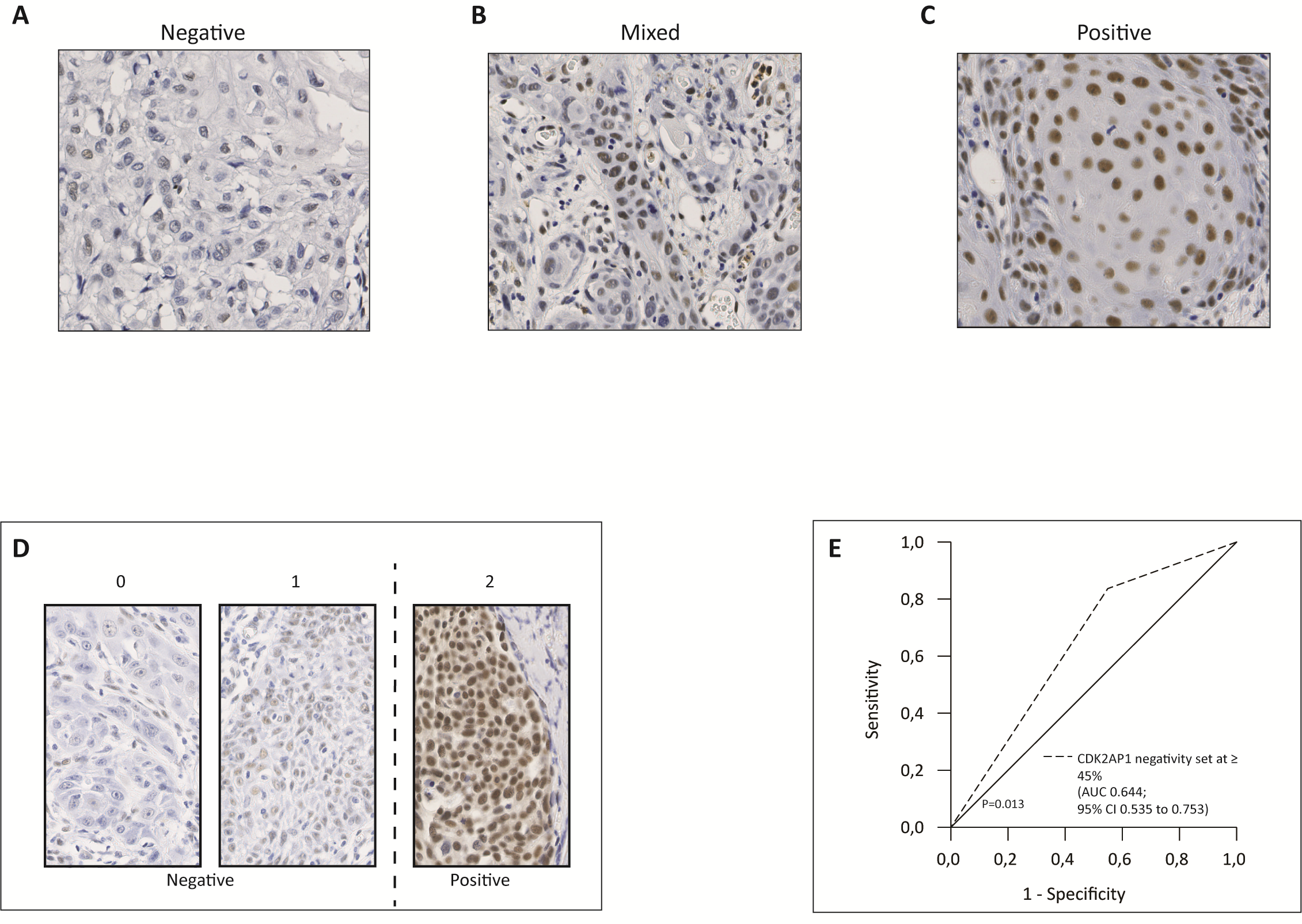


**Supplementary Figure 1. *CDK2AP1 IHC analysis in the OSCC patient cohort.***
**A-C.** Representative examples of CDK2AP1-negative (A), -mixed (B), and -positive (C) tumors as determined by IHC.
**D**. Representative examples of CDK2AP1 staining patterns as determined by IHC. Staining intensity 0 and 1 refer to CDK2AP1 low/negative tumors (<45% positive cells), whereas 2 indicates the fully positive cases (>45% positive cells).
**E.** The ROC (receiver operating characteristic) curve shows the optimal threshold of 45.0% of CDK2AP1-negative cells here employed for disease-free survival analysis.


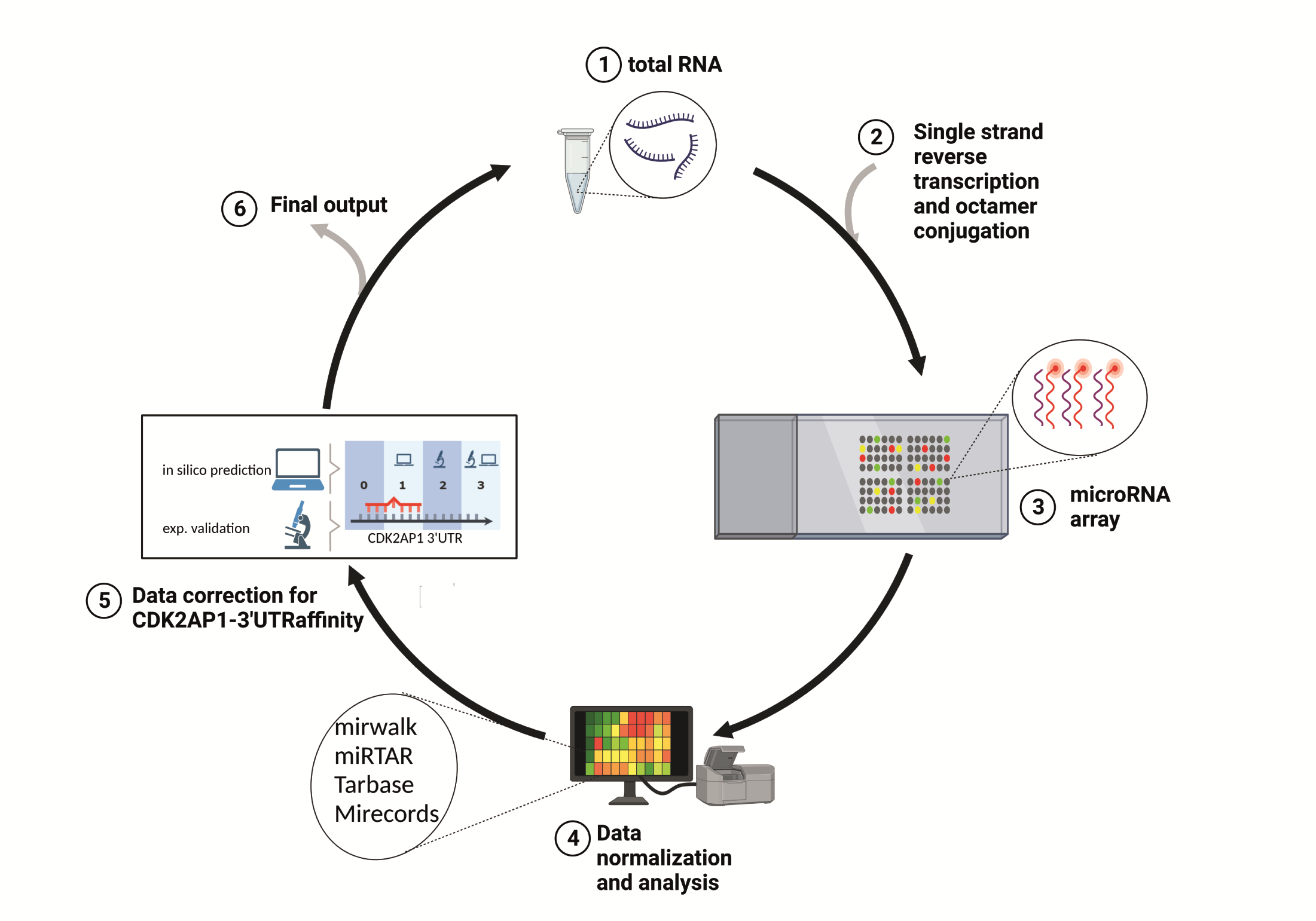


**Supplementary Figure 2. *Overall strategy for the screening and identification of*** ***CDK2AP1-antagonist miRNAs.***

Total RNA was isolated from the CDK2AP1-deficient OSCC cell lines panel (1) and employed to generate a cDNA library (2) as a probe for the microRNA arrays (3). A total of 1271 pre-miRs were originally identified by microarray profiling whose expression levels were subsequently normalized and compared with those reported in the publicly available Cancer Cell Line Encycolopedia (CCLE) database of head and neck SCC cell lines (4). The final rank of miR candidates was obtained by *in-silico* prediction and by experimental validation of their specific affinity with the 3’-UTR of *CDK2AP1* (5) (see Material and Methods and Supplementary Table 3).


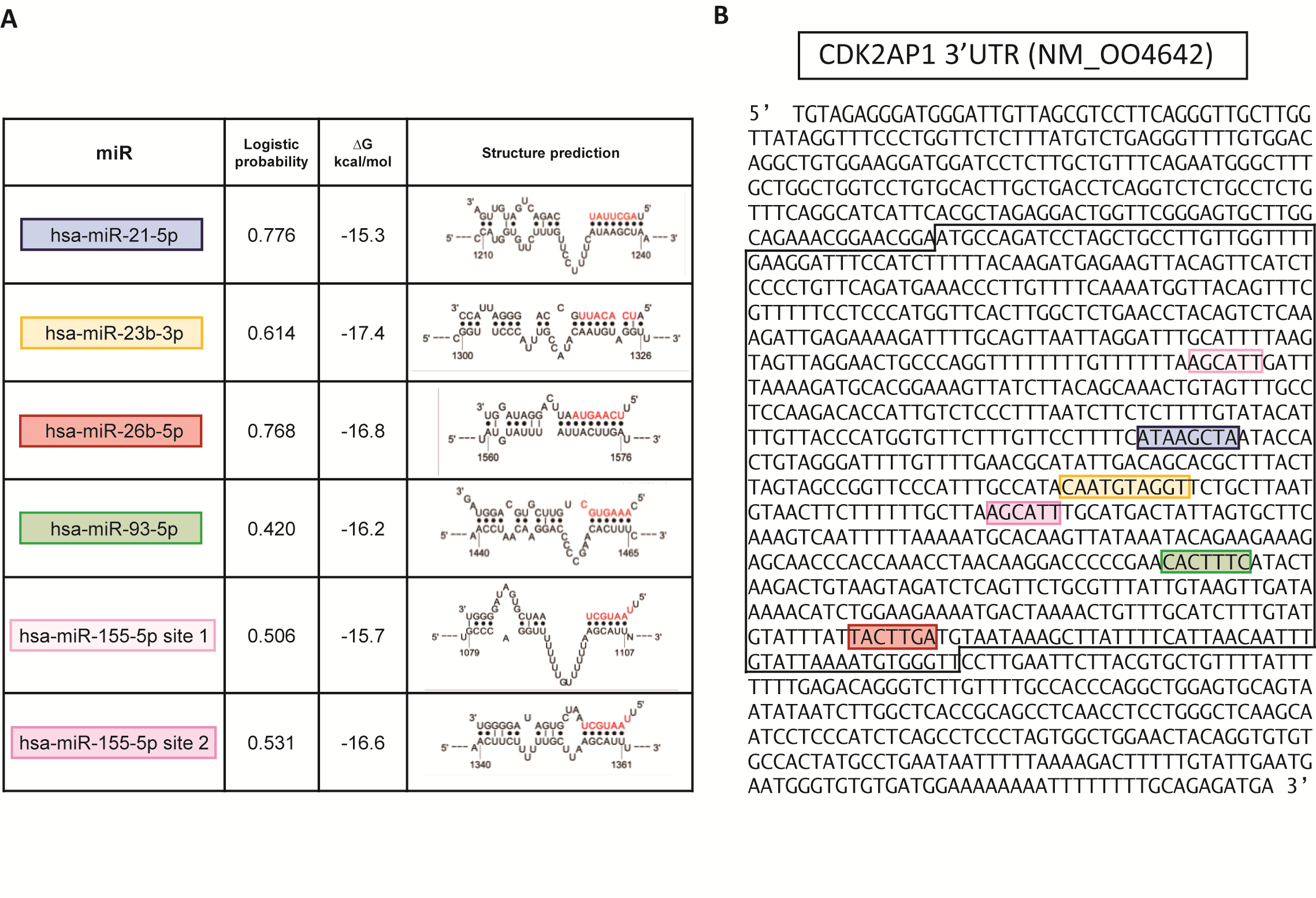


**Supplementary Figure 3. *StarMIr predictions of the interactions between the candidate miRs and the CDK2AP1 3’UTR.***

1. Logistic probability, total ∆G, and structural predictions generated by the StarMiR software are depicted for the interaction of each of the selected candidate microRNA with the 3’-UTR of *CDK2AP1*.
2. Genomic sequence of the last (4^th^) *CDK2AP1* exon (NM_004642) and its 3’-UTR (frame-delimited). The predicted seed sequences are depicted in colors corresponding to the selected miRs, as in A.


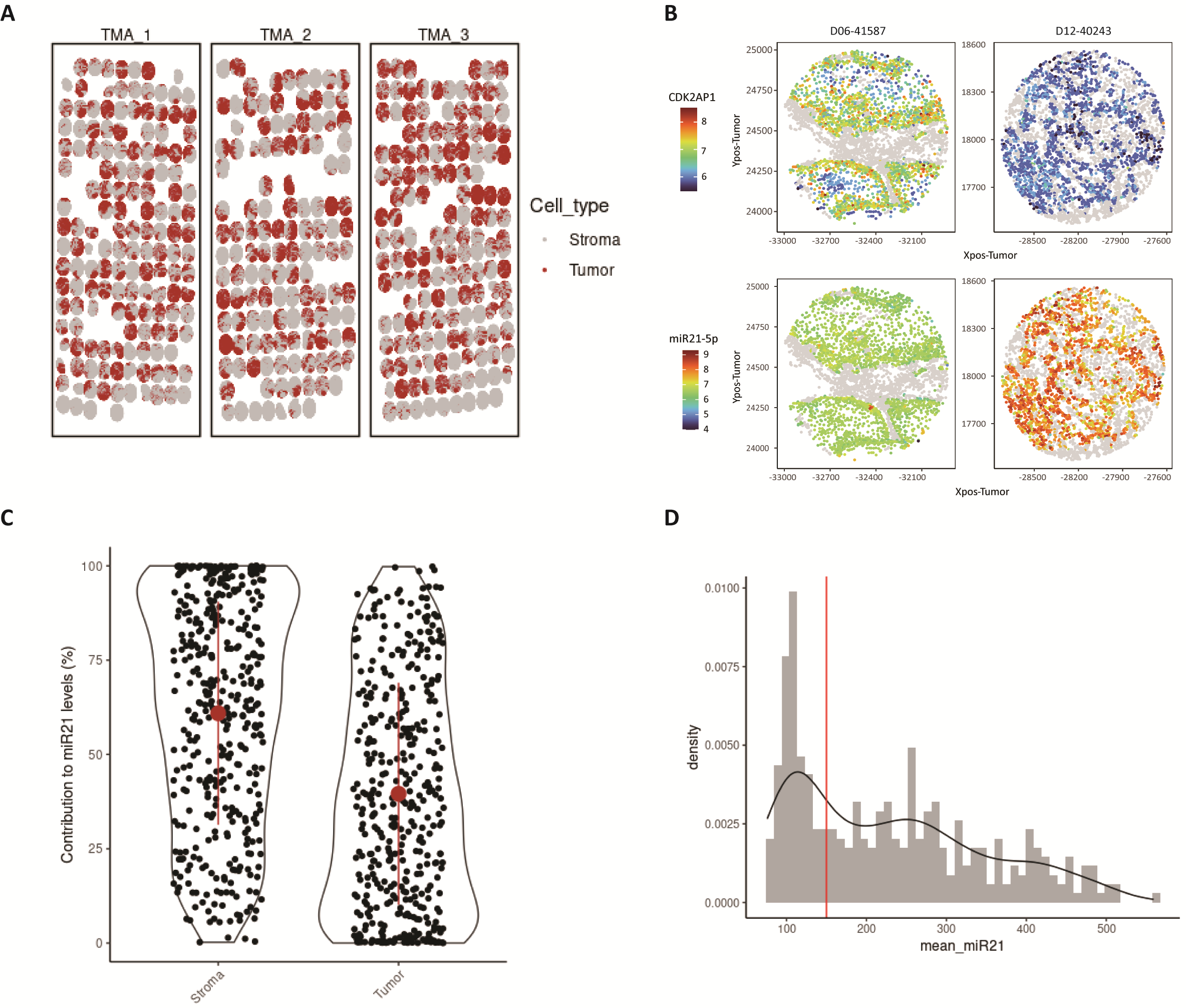


**Supplementary Figure 4. *Computational analysis of TMAs by combined IF and ISH.***

1. Digital reconstruction of the 3 TMAs encompassing a total of 432 tumor cores. For each core, the tumor cells (red dots) and stromal cells (grey dots) are displayed.

**B.** Digital reconstruction of the cores showed in Figure 5A, highlighting the expression densities of CDK2AP1 in the nucleus (upper panels) and miR-21-5p in the cytoplasm (lower panels) in the respective tumor fractions.

**C.** Violin plot showing the contribution for miR-21-5p levels in each core, stratified by stroma and tumor parenchyma.

**D.** Bar plot analysis of the core densities relative to miR-21-5p expression (intensity). The cores with a mean miR-21-5p intensity above 150 are classified as miR-21^high^, while the ones below 150 are miR21^low^.

**Supplementary Tables**

***Supplementary Table 1***. Taqman probe IDs employed for cDNA production and miRNA-RT-qPCR.

| *Assay name* | *miRBase Accession Number* | *Assay ID* |
| --- | --- | --- |
| hsa-miR-21-5p | MIMAT0000076 | 002438 |
| hsa-miR-23b-3p | MIMAT0000418 | 245306_mat |
| hsa-miR-26b-5p | MIMAT0000083 | 000407 |
| hsa-miR-93-5p | MIMAT0000093 | 001090 |
| hsa-miR-155-5p | MIMAT0000646 | 467534_mat |

***Supplementary Table 2***. List of oligonucleotides employed in the plasmids construction.

| *Oligo name* | *Sequence* | *RE* | *Purpose* |
| --- | --- | --- | --- |
| CDK2AP1_3UTR_F | 5’-ATGCTCTAGATGCCTTGTTGGTTTTGAAGG-3’ | XbaI | Clone CDK2AP1 3’UTR in pGL3 reporter. |
| CDK2AP1_3UTR_R | 5’-ATGCGGCCGGCCACATTTTAATACAAAT-3’ | FseI |  |
| Premir23b_F | 5’-GGCCTCTAGAGTTAGAGGCAGTAAACTAA-3’ | XbaI | Clone pre-miR sequence in PCDH expression vector. |
| Premir23b_R | 5’-ATAAGGATCCTCCAAAGAAGAGCTACGAG-3’ | BamHI |  |
| Premir26b_F | 5’-ATGGTCTAGACTTCATCATCCCTGTGGAG-3’ | XbaI |  |
| Premir26b_R | 5’-ATAAGGATCCTACCTCACGAGGTCCCTAA-3’ | BamHI |  |
| Premir93_F | 5’-ATCCTCTAGATAAAGTGCTGACAGTGCAG-3’ | XbaI |  |
| Premir93_R | 5’-ATACGGATCCAAGAAGACAATTGGCAGAG-3’ | BamHI |  |
| Premir155_F | 5’-GGCCTCTAGATTTACTATATGCTGTCACTC-3’ | XbaI |  |
| Premir155_R | 5’-GGACGGATCCTAGTTTAAGGTTGAACATC-3’ | BamHI |  |
| Muta_seed_21_F | 5’-CGATATACCACTGTAGGGATTTTGTTTTGAACG-3’ | - | Seed sequences mutagenesis. |
| Muta_seed_21_R | 5’-AATAGAAAAGGAACAAAGAACACCATGGGTAAC-3’ | - |  |
| Muta_seed_23b_F | 5’-GTTCTGCTTAATGTAACTTCTTTTTTG-3’ | - |  |
| Muta_seed_23b_R | 5’-TATGGCAAATGGGAACCG-3’ | - |  |
| Muta_seed_26b_F | 5’-TGTAATAAAGCTTATTTTCATTAAC-3’ | - |  |
| Muta_seed_26b_R | 5’-ATAAATACATACAAAGATGCAAAC-3’ | - |  |
| Muta_seed_93_F | 5’-ATACTAAGACTGTAAGTAGATCTCAGTTC-3’ | - |  |
| Muta_seed_93_R | 5’-TCGGGGGTCCTTGTTAGG-3’ | - |  |
| Muta_seed_155_1_F | 5’-GATTTAAAAGATGCACGG-3’ | - |  |
| Muta_seed_155_1_R | 5’-TAAAAAACAAAAAAAACCTGGG-3’ | - |  |
| Muta_seed_155_2_F | 5’-TGCATGACTATTAGTGCTTC-3’ | - |  |
| Muta_seed_155_2_R | 5’-TAAGCAAAAAAGAAGTTACATTAAG-3’ | - |  |
| PGL3_seq_1 | 5’-AACGCATATTGACAGCACGC-3’ | - | Sequencing pGL3 CDK2AP1 3’UTR insert |
| PGL3_seq_2 | 5’-TATCATGTCTGCTCGAAGCG-3’ | - |  |
| CMV_seq_1 | 5’-GGCACCAAAATCAACGGGAC-3’ | - | Sequencing PCDH vector |

***Supplementary Table 3***. Output of the analysis of miRNA microarray. (Available on “Supplementary Table 3_Stabile et al.xls”)
