# Supplementary figures and images for: The deleted in oral cancer (DOC1 aka CDK2AP1) tumor suppressor gene is downregulated in oral squamous cell carcinoma by multiple microRNAs
Source: Cell Death Dis. 2023 May 22;14(5):337. doi: 10.1038/s41419-023-05857-2 (PMC10202934; doi:10.1038/s41419-023-05857-2)

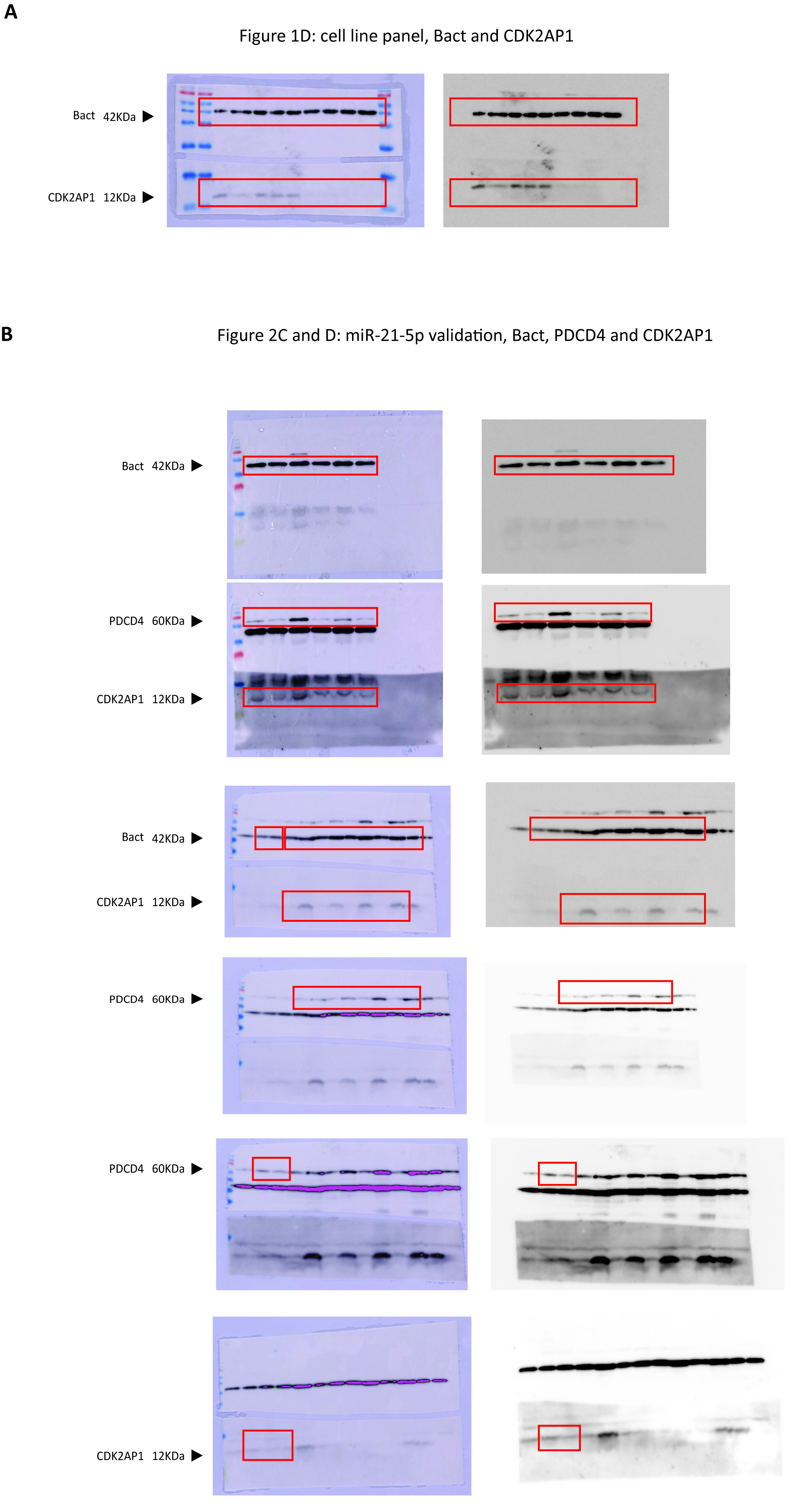

Supplement: Supplementary file 3 — Original Data File [file 41419_2023_5857_MOESM3_ESM.tif]
